# Long-term radiological progression after resection of dysembryoplastic neuroepithelial tumors: patterns and prognostic factors

**DOI:** 10.1007/s11060-026-05473-y

**Published:** 2026-04-07

**Authors:** Taehoon Kim, Seung-Ki Kim, Chun Kee Chung, Chul-Kee Park, Ki Joong Kim, Byung Chan Lim, Woojoong Kim, Joo Whan Kim, Ji Hoon Phi

**Affiliations:** 1 Division of Pediatric Neurosurgery, Seoul National University Children’s Hospital, Seoul, Republic of Korea; 2 Department of Neurosurgery, Seoul National University Hospital, Seoul, Republic of Korea; 3 Neuroscience Research Institute, Seoul National University Medical Research Center, Seoul, Republic of Korea; 4 Department of Pediatrics, Seoul National University Children’s Hospital, Seoul, Republic of Korea; 5 Division of Pediatric Neurosurgery, Seoul National University Children’s Hospital Seoul National University College of Medicine, 101 Daehak-ro, Jongno-gu, Seoul, 03080 Republic of Korea

**Keywords:** Dysembryoplastic neuroepithelial tumor, Gross total resection, Progression-free survival, Postoperative complications

## Abstract

**Purpose:**

To characterize long-term radiological progression after resection of dysembryoplastic neuroepithelial tumors (DNETs) and identify prognostic factors, focusing on satellite lesions (SLs) and extent of resection.

**Methods:**

We retrospectively reviewed 84 patients with pathologically confirmed DNETs who underwent surgery at a single institution. Tumor location and satellite lesions (SLs) were assessed on preoperative MRI. Tumor progression-free survival (PFS) was analyzed using Kaplan–Meier analysis with log‑rank tests and Cox regression, and seizure recurrence was compared using Fisher’s exact test.

**Results:**

Over a median follow-up of 9.3 years, 24 patients (29%) demonstrated radiological progression. No progression occurred after gross total resection (GTR) (0/43), whereas 24/41 (59%) progressed after non-GTR (10-year PFS, 100% vs. 33.7%). SL-positive tumors showed higher progression than SL-negative tumors (44% vs. 10%) and remained independently associated with progression (hazard ratio [HR], 4.89; 95% CI, 1.57–15.23). Younger age at surgery and central lobe involvement were additional independent predictors. Seizure recurrence was more frequent in patients with radiological progression (67% vs. 20%).

**Conclusion:**

GTR provides excellent long‑term tumor control in DNET. When GTR is not achievable, SLs and central lobe involvement—particularly in younger patients—identify a high‑risk subgroup requiring maximal safe resection and close surveillance.

**Supplementary Information:**

The online version contains supplementary material available at 10.1007/s11060-026-05473-y.

## Introduction

Dysembryoplastic neuroepithelial tumors (DNETs) are benign glioneuronal neoplasms first described in 1988 [1]. They most commonly present with focal seizures that may become medically intractable during childhood or adolescence, making DNETs a prototype example of long-term epilepsy-associated tumors (LEATs) [2–5]. Surgical resection is performed to achieve seizure control and prevent tumor growth.

Although early series suggested that DNET could be cured by surgery, subsequent reports have shown that gross total resection (GTR) is not always feasible and that residual tumor may progress, necessitating reoperation [1, 6–9]. Achieving GTR can be particularly challenging for lesions involving eloquent cortex, including the central lobe, because aggressive resection may increase the risk of permanent neurological deficits [10].

Satellite lesions (SLs)—small, separate foci of tumor adjacent to the main lesion—have been increasingly recognized on MRI in patients with DNET [11–13]. Prior studies have suggested that SLs may be associated with incomplete resection and higher recurrence rates, but their prognostic value and impact on long‑term radiological progression remain uncertain [7–9, 11–13].

In parallel, Chassoux et al. at Sainte‑Anne Hospital proposed an MRI‑based classification that stratifies DNETs into three patterns—type I (cystic), type II (nodular), and type III (dysplastic)—and showed correlations with histologic patterns, tumor location, and extent of the epileptogenic zone [14]. However, the prognostic value of this scheme for tumor progression and seizure outcome has not been consistently validated in independent cohorts.

Based on these observations, we analyzed a consecutive cohort of patients with histologically confirmed DNET treated at a single institution. Our primary objective was to characterize long‑term tumor control after surgical resection of DNET and to identify clinical and anatomical factors associated with radiological progression. As a secondary aim, we examined patterns of incomplete resection and their relationship to tumor location and SLs and descriptively explored how progression related to seizure recurrence and reoperation.

## Materials and methods

### Patient inclusion

We identified consecutive patients who underwent surgical resection for brain tumors and were diagnosed with DNET at Seoul National University Children’s Hospital and Seoul National University Hospital between January 2001 and January 2024. All cases were histologically confirmed as DNET. Patients with prior tumor surgery at outside institutions were not included. Both pediatric and adult patients were included. This study was approved by the Institutional Review Board of Seoul National University Hospital (IRB No. 2410-161-1581); the requirement for informed consent was waived owing to the retrospective design.

### Data acquisition and radiologic review

Clinical variables (demographics, epilepsy history, and operative details) were extracted from medical records. Preoperative MRI was reviewed to determine tumor location (frontal, temporal, parietal, central, or occipital lobe). The central lobe (sensory–motor cortex: precentral, postcentral, and paracentral gyri) was defined according to Yaşargil and considered a high‑risk area for postoperative neurological deficits [10].

SLs were defined as small nodular or crescent foci adjacent to the main mass but separated from it by intervening normal parenchyma on preoperative MRI [11–13]. Lesions were classified according to the Sainte-Anne MRI system (types 1–3) [14]. When available, molecular alterations relevant to contemporary diagnostic criteria (e.g., FGFR1 alterations) were recorded [15–17].

### Surgical strategy and extent of resection

All procedures were performed with the intent of achieving maximal safe resection. Extent of resection (EOR) was determined on early postoperative MRI (obtained within 48 h after surgery in all patients) and categorized as GTR (no residual main lesion or SLs) or non-GTR (any residual main lesion and/or unresected SLs). Reoperations during follow‑up were recorded. For descriptive analyses of clinical trajectories, the EOR after the last surgery was similarly classified and referred to as final resection.

### Follow-up and outcome measurement

Radiological progression was defined as the appearance of a new lesion at or near the resection cavity or interval growth of a residual lesion on serial MRI. Progression-free survival (PFS) was calculated from the date of initial surgery to radiological progression or the last imaging follow-up. Seizure outcome was assessed at 12 months and at the last follow-up using the Engel classification; Engel class was determined by chart abstraction of postoperative seizure history documented in outpatient records. Seizure recurrence was defined as any postoperative unprovoked seizure and dichotomized as Engel class I versus classes II–IV for descriptive analyses. Postoperative complications were recorded and categorized as transient or permanent.

### Statistical analysis

Statistical analyses were performed using RStudio (version 2025.05.0 Build 496). Continuous variables are presented as median (range) or mean (standard deviation), and categorical variables as counts and percentages.

PFS curves were estimated using the Kaplan–Meier method and compared between groups (GTR vs. non-GTR, SL present vs. absent, tumor location) with log‑rank tests. Time‑to‑event associations were quantified with Cox proportional hazards models and summarized as hazard ratios (HRs) with 95% confidence intervals (CIs). Univariable Cox models were first fitted for candidate predictors including age at surgery, sex, central lobe involvement of tumor, SL presence, Sainte‑Anne MRI type, and EOR. Variables with *p* < 0.05 in univariable analyses were considered for multivariable modeling. Because no progression occurred after GTR, EOR could not be included in multivariable Cox models. We therefore fitted a model including age and sex (forced covariates) together with central lobe location and SL presence, which were significant in univariable analysis, to assess whether these variables were independently associated with PFS.

The association between tumor progression and seizure recurrence was assessed using Fisher’s exact test. Recurrence patterns (no recurrence, tumor progression only, seizure recurrence only, or both), reoperation (yes/no), and final Engel class were summarized descriptively and visualized as an alluvial diagram. Proportions of postoperative complications were summarized per procedure and per patient, and 95% CIs for these proportions were obtained using exact binomial methods. All statistical tests were two‑sided, and *p* < 0.05 was considered statistically significant.

## Results

### Patient demographics and clinical characteristics

A total of 84 patients were included (Table 1). Median age at surgery was 12.9 years (range, 1.0–57.2 years), and 53 patients (63%) were male. Tumors most commonly arose in the temporal lobe (43/84, 51%), followed by the frontal (17/84, 20%), central (11/84, 13%), parietal (9/84, 11%), and occipital (4/84, 5%) lobes. SLs were identified on preoperative MRI in 45 patients (54%) and were particularly frequent in central and parietal tumors (Online Resource 1). Sainte‑Anne MRI types and available molecular alterations are summarized in Table 1.

**Table 1:** Baseline characteristics of 84 patients with dysembryoplastic neuroepithelial tumors and crude rates of radiological tumor progression

Variables	Numbers (%)	Tumor progression (%)
Median age (range)		
At surgery	12.9 (1.0-57.2)	
At epilepsy onset	11.7 (0.4–47.3)	
Sex		
Male	53 (63)	18 (34)
Female	31 (37)	6 (19)
Duration of epilepsy		
Mean (SD, months)	28.4 (55)	
< 6 months	39 (46)	11 (28)
< 2 months	25 (30)	6 (24)
Location		
Temporal	43 (51)	5 (12)
Central	11 (13)	9 (82)
Frontal	17 (20)	4 (24)
Parietal	9 (11)	6 (67)
Occipital	4 (5)	0 (0)
Extent of resection		
GTR	43 (51)	0 (0)
Non-GTR	41 (49)	24 (59)
SL		
Present	45 (54)	20 (44)
Absent	39 (46)	4 (10)
Engel classification at first year		
I	75 (89)	
II-IV	9 (11)	
Engel classification at the last follow-up		
I	73 (87)	
II-IV	11 (13)	
Sainte-Anne MR classification		
Type 1	60 (71)	19 (32)
Type 2	3 (4)	0 (0)
Type 3	21 (25)	5 (24)
Genetic alteration (*n* = 16)		
FGFR1 mutation	8 (50)	4 (50)
FGFR1 TKDD	6 (38)	1 (17)
FGFR1 fusion	2 (12)	1 (50)

Values are given as number of patients (%) unless otherwise indicated. Tumor progression (%) indicates the proportion of patients in each category who developed radiological progression during follow‑up

FGFR1 = fibroblast growth factor receptor 1; GTR=gross total resection; MR=magnetic resonance imaging; SD=standard deviation; SL=satellite lesion; TKDD=tandem kinase-domain duplication

Early postoperative MRI demonstrated GTR in 43/84 patients (51%) and non‑GTR in 41/84 (49%). Among the 41 patients with non‑GTR, 26 had residual main tumor, whereas in 15 the main mass was completely removed but SLs were left in situ. Of the 45 patients with SLs, GTR including complete removal of all SLs was achieved in 13 (29%), while the remaining 32 (71%) had non‑GTR. At 12 months, 75/84 patients (89%) were Engel class I; at last follow‑up, 73/84 (87%) remained Engel class I (Table 1).

### Tumor progression and extent of resection

Over a median follow-up of 9.3 years (range, 0.6–22.8 years), 24 patients (29%) demonstrated radiological progression. Five- and 10-year PFS rates were 73.8% and 65.7%, respectively. Progression occurred most often in central (9/11, 82%), parietal (6/9, 67%), and frontal (4/17, 24%), compared with temporal (5/43, 12%) and occipital (0/4, 0%) (Table 1). Patients with SLs had higher crude progression rates (20/45, 44%) than those without SLs (4/39, 10%).

**Fig. 1:**
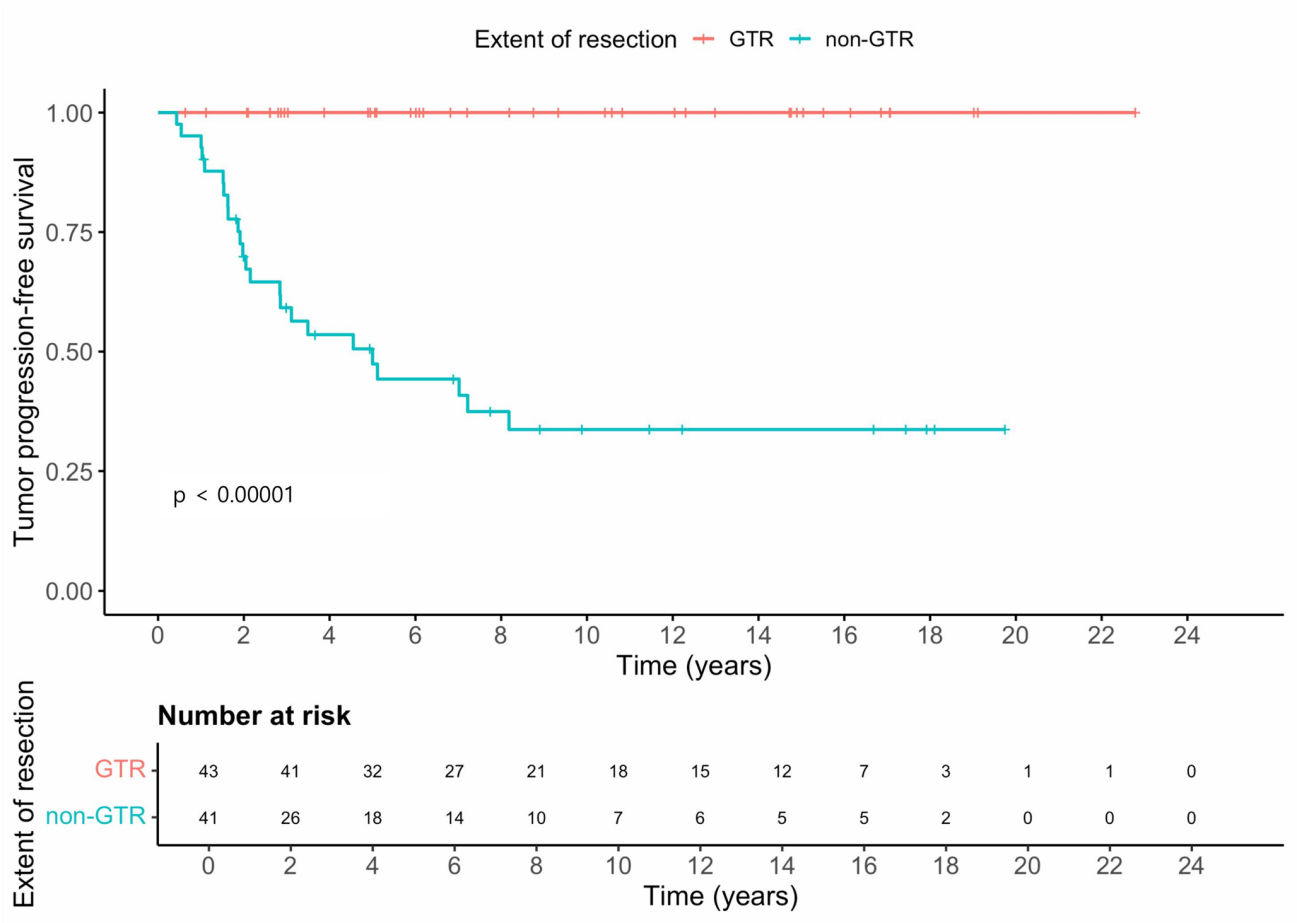
Tumor progression‑free survival according to extent of resection. Kaplan–Meier curves compare PFS between patients who achieved gross total resection (GTR, *n* = 43) and those with non‑GTR (*n* = 41). No radiological tumor progression occurred after GTR, whereas most events arose in the non‑GTR group (log‑rank *p* < 0.00001). The estimated 5‑/10‑year PFS was 100.0% (95% CI 100.0–100.0)/100.0% (95% CI 100.0–100.0) in the GTR group and 47.4% (95% CI 33.7–66.7)/33.7% (95% CI 20.8–54.5) in the non‑GTR group. GTR = gross total resection

No progression occurred after GTR (0/43), whereas 24/41 (59%) progressed after non-GTR (log-rank *p* < 0.00001; Fig. 1). Because of complete separation, the HR for non-GTR versus GTR was not estimable.

Central‑lobe tumors and SL‑positive tumors were less likely to undergo GTR. Only 2/11 central tumors (18%) achieved GTR compared with 41/73 non‑central tumors (56%). Among patients with SLs, complete removal of the main mass and all SLs (GTR) was achieved in 13/45 (29%), whereas 30/39 patients without SLs (77%) underwent GTR. These patterns suggest that central location and SLs are major practical reasons why GTR could not be obtained.

### Predictors of tumor progression

**Fig. 2:**
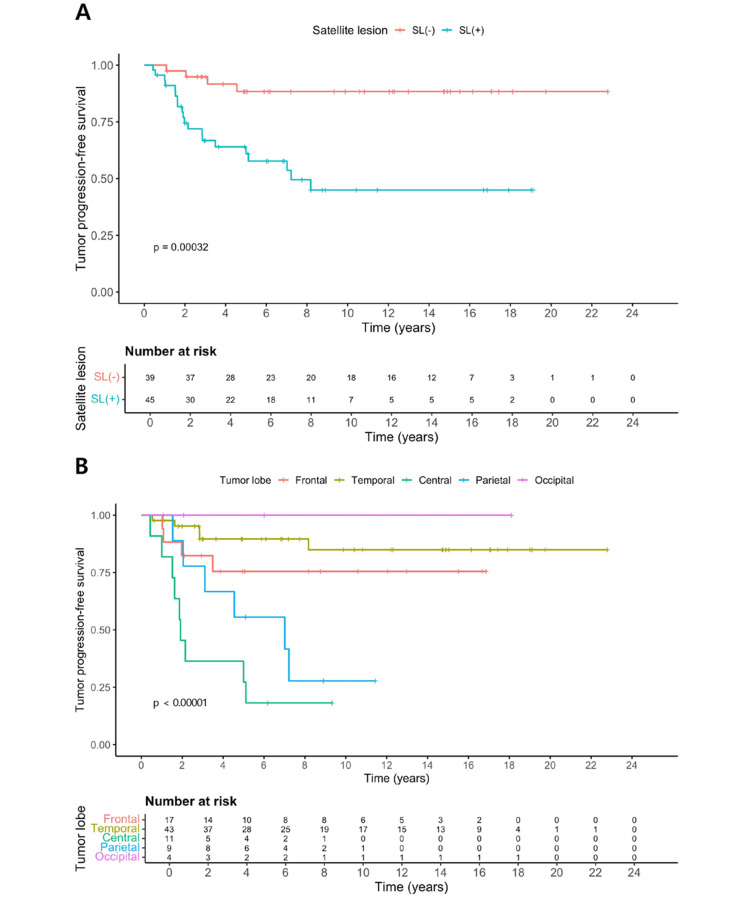
Tumor progression‑free survival according to SL status and tumor location. (**A**) Kaplan–Meier curves for progression‑free survival stratified by presence (SL+, *n* = 45) or absence (SL–, *n* = 39) of SLs (log‑rank *p* = 0.00032). The estimated 5‑/10‑year PFS was 60.9% (95% CI 47.5–78.2)/45.0% (95% CI 30.4–66.6) for SL(+) and 88.4% (95% CI 78.2–99.9)/88.4% (95% CI 78.2–99.9) for SL(–). (**B**) Kaplan–Meier curves for PFS by tumor lobe (frontal, temporal, central, parietal, occipital; global log‑rank *p* < 0.00001). The estimated 5‑/10‑year PFS was frontal 75.5% (95% CI 57.1–99.7)/75.5% (95% CI 57.1–99.7), temporal 89.6% (95% CI 80.5–99.8)/84.9% (95% CI 73.0–98.8), central 27.3% (95% CI 10.4–71.6)/18.2% (95% CI 5.2–63.7), parietal 55.6% (95% CI 31.0–99.7)/27.8% (95% CI 8.9–86.9), and occipital 100.0% (95% CI 100.0–100.0)/100.0% (95% CI 100.0–100.0). Central and parietal tumors showed the poorest PFS. SL = satellite lesion

KM curves stratified by SL status underscored the impact of tumor morphology on PFS. SL-positive tumors showed significantly worse PFS than SL-negative tumors (log-rank *p* = 0.00032; Fig. 2A). By lobe, central and parietal tumors had the poorest PFS, whereas temporal and occipital tumors had the most favorable course (overall log‑rank *p* < 0.00001; Fig. 2B).

In univariable Cox models, non-GTR (vs. GTR), younger age at surgery, central lobe involvement, and SL presence were associated with progression, whereas sex and Sainte‑Anne MRI type were not (Table 2). Age at surgery had an HR of 0.89 per year (95% CI 0.83–0.95; *p* = 0.00087), corresponding to an approximately 11% relative reduction in risk per additional year. Central lobe involvement (HR 6.69, 95% CI 2.88–15.54; *p* = 0.00001) and SL presence (HR 5.75, 95% CI 1.96–16.86; *p* = 0.001) also predicted higher risk.

**Table 2:** Univariable and multivariable Cox proportional‑hazards analyses for tumor progression‑free survival

	Univariable analysis		Multivariable analysis	
	HR (95% CI)	*P* value	HR (95% CI)	*P* value
Age at surgery	0.89 (0.83–0.95)	0.00087	0.90 (0.83–0.97)	0.00748
Sex	1.85 (0.73–4.66)	0.19341	0.94 (0.35–2.49)	0.89509
Central lobe	6.69 (2.88–15.54)	0.00001	2.85 (1.11–7.28)	0.02872
Sainte-Anne MR type	0.65 (0.24–1.75)	0.39569		
Presence of satellite lesions	5.75 (1.96–16.86)	0.00144	4.89 (1.57–15.23)	0.00617
Gross total resection	NE			

CI=confidence interval; GTR=gross total resection; HR=hazard ratio; MR=magnetic resonance imaging; NE = not estimable; SL=satellite lesion. NE denotes that the HR could not be estimated because no progression events occurred after GTR

Candidate predictors included age at surgery, sex, central lobe involvement, Sainte‑Anne MRI type, SL presence, and GTR versus non‑GTR. The multivariable model included age at surgery, sex, central lobe location, and SL presence. Hazard ratios (HRs) are shown with 95% confidence intervals (CIs). No tumor progression occurred after GTR, so HRs for non‑GTR versus GTR could not be reliably estimated (NE)

Given complete separation for EOR, we fitted a multivariable Cox model including age and sex (forced) plus central lobe involvement and SL presence (univariable *p* < 0.05). Younger age remained independently associated with progression (HR 0.90 per year, 95% CI 0.83–0.97; *p* = 0.007), as did central lobe involvement (HR 2.85, 95% CI 1.11–7.28; *p* = 0.029) and SL presence (HR 4.89, 95% CI 1.57–15.23; *p* = 0.006). Sex was not associated with progression (HR 0.94, 95% CI 0.35–2.49; *p* = 0.895) (Table 2).

Overall, GTR was the dominant determinant of tumor control, while younger age, central lobe involvement, and SL presence emerged as key adverse features. These factors were associated with worse PFS and, for central lobe tumors and SL‑positive lesions, a lower likelihood of achieving GTR.

### Seizure outcomes and their relationship to tumor control

Seizure outcomes were generally favorable but closely tied to tumor control. Engel class I was achieved in 75/84 patients (89%) at 1 year and 73/84 (87%) at last follow‑up (Table 1); 28/84 (33%) experienced postoperative seizure recurrence.

**Fig. 3:**
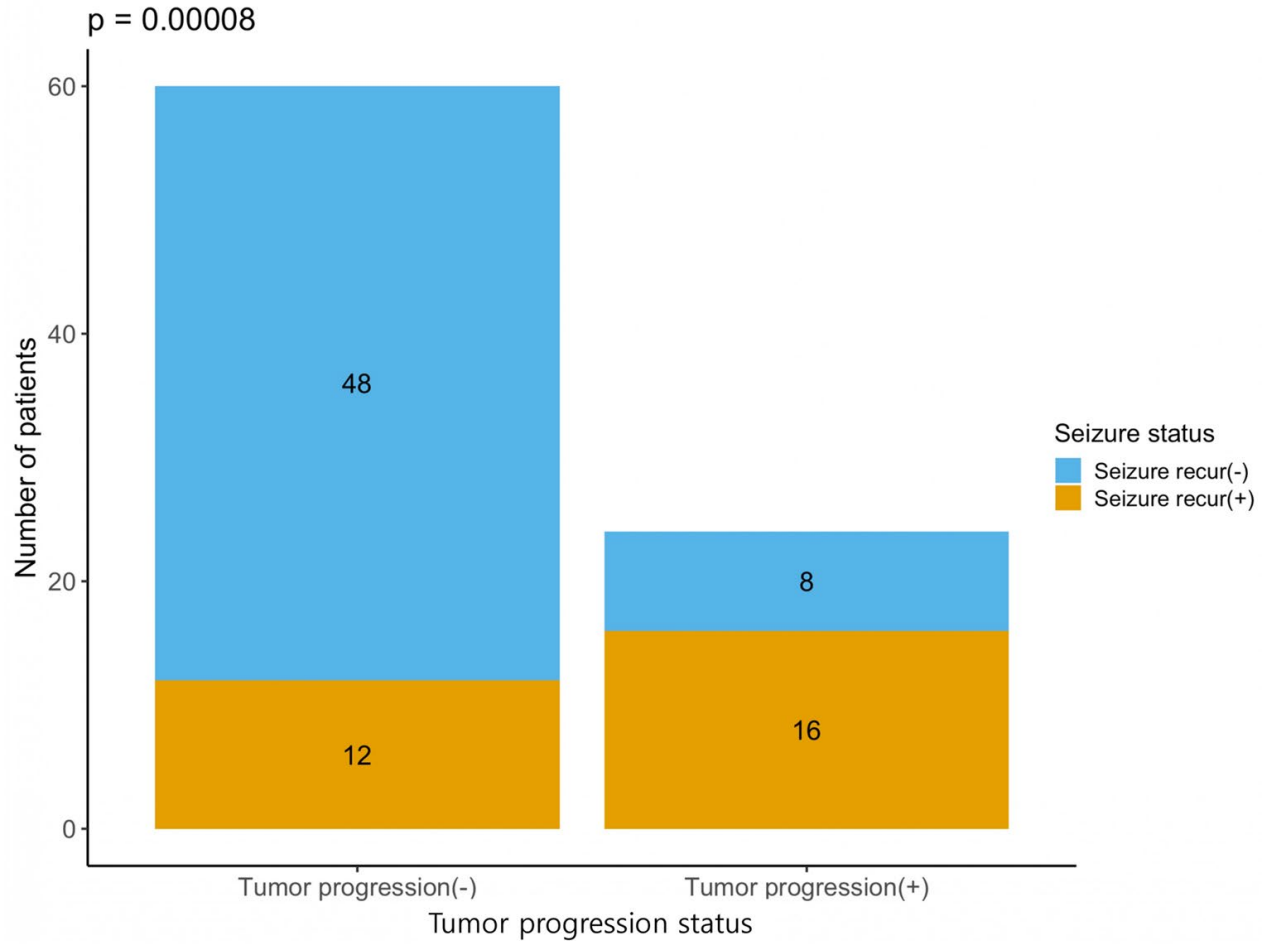
Association between tumor progression and postoperative seizure recurrence. Stacked bar chart showing the joint distribution of tumor progression status (progression– vs. progression+) and seizure outcome (seizure recurrence– vs. seizure recurrence+). Seizure recurrence occurred in 16/24 patients (67%) with tumor progression versus 12/60 (20%) without progression. Fisher’s exact test demonstrated a strong association between tumor progression and seizure relapse (*p* = 0.00008)

Seizure recurrence was markedly more frequent with tumor progression (16/24 [67%]) than without progression (12/60 [20%]; OR 8.0, Fisher’s exact *p* = 0.00008; Fig. 3).

Patients without tumor or seizure recurrence rarely required reoperation and predominantly remained Engel class I, whereas those with both tumor progression and seizure recurrence more often underwent reoperation and were over‑represented among Engel class II–IV outcomes (Online Resource 2). Among initially non‑GTR cases, conversion to GTR at reoperation tended to coincide with Engel class I at last follow‑up, while persistent non‑GTR was more often associated with ongoing seizures. These patterns are descriptive and should be interpreted cautiously, but they align with the PFS analyses, underscoring the link between durable tumor control and long‑term seizure freedom.

### Surgical complications

Across 102 surgeries in 84 patients, 14 permanent neurological deficits occurred (13.7% per procedure; 95% CI 7.7–22.0), affecting 13 patients (15.5% per patient); 13 additional deficits were transient. The most frequent permanent deficits were hemiparesis (4 procedures), quadrantanopsia (6), and hemianopsia (2), followed by anosmia (1) and facial palsy (1) (Online Resource 3). Hemiparesis occurred mainly after central lobe resections, whereas permanent visual field deficits were most often associated with temporal and parietal resections.

Non‑neurological complications included 3 wound infections, 4 postoperative hemorrhages, and 1 wrong‑gyrus resection. Most complications occurred during attempts to maximize resection in or near eloquent cortex, indicating that while GTR provided excellent tumor control, it carried a meaningful risk of permanent neurological morbidity, particularly for lesions involving the central lobe and optic pathways.

## Discussion

In this single-center cohort of 84 patients with DNET and long follow-up (median 9.3 years), which represents one of the larger single-institution series with systematically reviewed imaging and surgical outcomes, the extent of resection and a small set of anatomical features largely determined long-term behavior. Overall, 29% developed radiological progression, but none recurred after GTR (0/43) compared with 59% after non‑GTR (24/41). Seizure recurrence clustered in the same subgroup, and ~ 14% of procedures resulted in permanent neurological deficits, mainly after resections involving sensorimotor cortex or visual pathways. Thus, GTR appears to provide durable tumor control, whereas the key clinical problem lies in patients in whom GTR cannot be safely achieved.

Our findings reinforce the importance of maximal resection in DNET. Prior series report excellent seizure outcomes and low recurrence after GTR, with ~ 70–85% achieving long‑term Engel class I after complete removal [3, 6–8, 11]. Consistent with this, no radiological progression occurred after GTR in our cohort, supporting that residual tumor should be considered a potential source of late regrowth rather than a harmless remnant [1].

SLs behaved as clinically relevant tumor burden rather than incidental imaging findings. SLs were present in more than half of patients, especially in central and parietal tumors, and were associated with worse PFS even after accounting for age and location. Together with prior work linking incomplete SL removal to higher recurrence and seizure relapse, these data support treating SLs as true tumor foci [11]. Genomic analyses align with this. Main masses and SLs share FGFR1 alterations yet show partially divergent trajectories, consistent with multifocal nodules from a common driver clone [18]. Thus, SLs should be managed as part of the neoplastic burden in surgical planning.

Central lobe tumors formed a particularly unfavorable subgroup. They were less likely to undergo GTR, more likely to progress, and almost always co‑occurred with SLs (9/11; Online Resource 1). This is anatomically plausible because the precentral, postcentral, and paracentral gyri subserve primary motor and somatosensory function, making aggressive resection high risk for permanent deficit. Surgeons therefore often accept non‑GTR once functional boundaries are reached, leaving presumed normal cortex or deep‑seated SLs in situ. This “central + SL‑positive” phenotype was technically difficult to clear and prone to subsequent progression despite low‑grade histology.

Within the broader LEAT spectrum, the central/parietal, SL‑rich phenotype appears anatomically constrained; thus, the higher-risk course in our cohort likely reflects limited resectability and residual SL burden rather than intrinsically aggressive biology [5, 19–21].

Younger age at surgery was also associated with higher progression risk. Rather than implying biological aggressiveness, this may reflect longer observation time and greater constraints on resection in very young children (more extensive dysplastic cortex, heavier SL burden, and limited functional mapping) [22–24]. Although speculative given the number of events, these considerations support closer surveillance in younger patients, particularly when SLs or central‑lobe involvement are present.

Seizure outcomes were generally favorable but closely linked to tumor control. Engel class I was maintained in 87% at last follow‑up, comparable to prior DNET series [3, 6–8, 11]. Seizure recurrence occurred in two‑thirds of patients with progression versus one‑fifth without, an approximately eight‑fold difference in odds. Figure 3 and Online Resource 2 show that progression and seizure recurrence cluster in the same subset, who more often undergo reoperation and are over‑represented among Engel class II–IV outcomes. Although we did not model seizure predictors, these patterns support that durable seizure freedom depends on durable tumor control.

Aggressive resection carried non‑negligible morbidity. Permanent neurological deficits occurred after 14 of 102 procedures (13.7%), most commonly hemiparesis after central lobe resections and visual field deficits after temporal/parietal resections involving optic radiations. Reoperation was performed in 14 patients and occurred only in those with radiological progression and/or seizure recurrence; it was most common when both were present (8/16) compared with progression alone (2/8) or seizures alone (4/12). Although the optimal surveillance schedule is undefined, these patterns support close MRI and epilepsy follow‑up for non‑GTR and/or SL‑positive patients—particularly younger children—with a low threshold for repeat imaging when seizures recur (e.g., MRI at 6–12 months postoperatively and then annually while residual disease persists). Reoperation may be considered for documented radiological progression of residual lesions even without seizures, and more strongly when progression coincides with recurrent seizures, while balancing functional risk. Overall, our data support maximal safe resection—pursuing GTR (including SLs) when feasible but accepting non‑GTR when functional risk is prohibitive—paired with close follow‑up and consideration of reoperation for progression and/or persistent seizures, consistent with broader LEAT experience [3–6].

Sainte‑Anne MRI type showed limited prognostic value. Most tumors were type I, and MRI type was not associated with PFS in univariable analysis. We therefore view the classification as useful for descriptive reporting but not as a primary determinant of strategy or risk [14].

Integrating these observations, we propose two broad surgical phenotypes. A favorable phenotype comprises mainly temporal (and some occipital) SL‑negative lesions, in which GTR is usually feasible and long‑term cure is common with low progression risk. A higher‑risk phenotype includes central or parietal SL‑positive tumors, often in younger patients, in whom GTR is frequently not achievable; progression and seizure relapse are common and reoperation may be required. This exploratory anatomical–radiologic stratification may aid preoperative counselling, surveillance planning, and decisions about staged or repeat surgery.

Limitations of this study include its retrospective, single-center design, evolving imaging/surgical practices over the long study period, and incomplete molecular profiling in the overall cohort; additionally, the absence of progression events after GTR precluded multivariable modeling that included EOR.

## Supplementary Information

Below is the link to the electronic supplementary material.

Supplementary Material 1

## Data Availability

De-identified data underlying this article will be shared on reasonable request to the corresponding author, subject to Institutional Review Board approval and applicable privacy regulations.
