# Supplementary material for: Long-term radiological progression after resection of dysembryoplastic neuroepithelial tumors: patterns and prognostic factors
Source: J Neurooncol. 2026 Apr 7;177(2):94. doi: 10.1007/s11060-026-05473-y (PMC13056769; doi:10.1007/s11060-026-05473-y)
Supplement: Supplementary file 1 — Supplementary Material 1 [file 11060_2026_5473_MOESM1_ESM.docx]

**
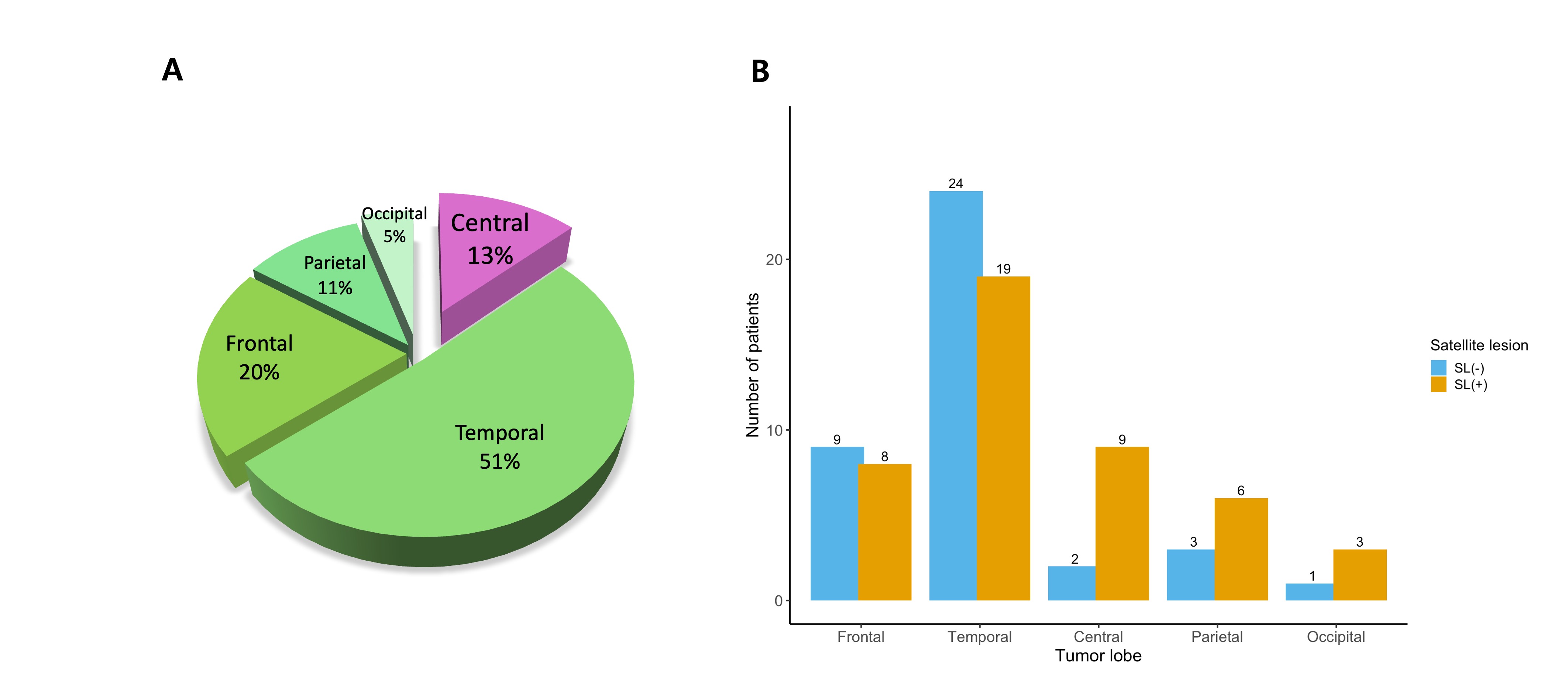
Online Resource captions**

**Online resource 1** Tumor location and distribution of satellite lesions. (A) Pie chart showing the lobar distribution of 84 DNETs (temporal 51%, frontal 20%, parietal 11%, central 13%, occipital 5%). (B) Grouped bar chart showing the number of patients with and without SLs in each lobe. SLs were most frequent in temporal, central, and parietal tumors.

**
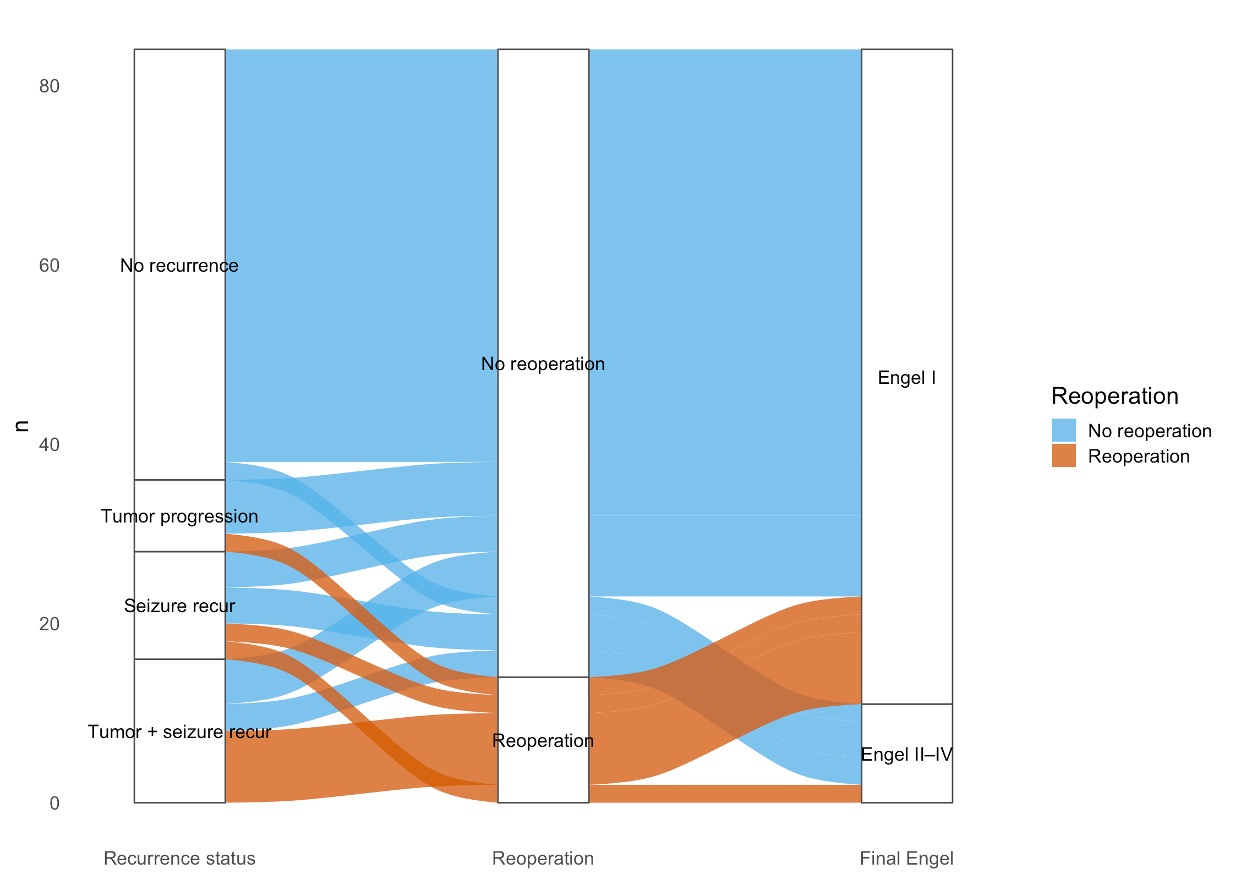
Online resource 2** Patient trajectories linking recurrence pattern, reoperation, and final seizure outcome. Alluvial diagram depicting flows from recurrence status (no recurrence, tumor progression only, seizure recurrence only, or both tumor and seizure recurrence) to reoperation status (no reoperation vs reoperation) and final Engel class (Engel I vs Engel II–IV). Stream width is proportional to the number of patients. Orange streams denote patients who underwent reoperation; blue streams denote those managed without reoperation. The figure highlights that most patients without recurrence remained Engel I without reoperation, whereas tumor and/or seizure recurrence concentrated among those who underwent reoperation and among final Engel II–IV outcomes

|  | Transient | Permanent | Total | Lobe |
| --- | --- | --- | --- | --- |
| Hemiparesis | 6 | 4 | 10 | Central(8), Temporal(2) |
| Quadrantanopsia | 1 | 6 | 7 | Temporal(5), Parietal(1), Occipital(1) |
| Hemianopsia | 1 | 2 | 3 | Temporal(2), Occipital(1) |
| Anosmia |  | 1 | 1 | Frontal(1) |
| Facial palsy | 3 | 1 | 4 | Central(2), Temporal (1), Frontal(1) |
| 3^rd^ nerve palsy | 1 |  | 1 | Temporal (1) |
| 4^th^ nerve palsy | 1 |  | 1 | Temporal (1) |
| Infection |  |  | 3 | Temporal (2). Parietal(1) |
| Hemorrhage |  |  | 4 | Temporal (4) |
| Wrong gyrus |  |  | 1 | Frontal(1) |

**Online resource 3** Postoperative complications after 102 surgical procedures in 84 patients with dysembryoplastic neuroepithelial tumors. Complications are listed by type and lobe involved and are classified as transient or permanent. Overall, 14 permanent neurological deficits occurred (13.7% per procedure; 95% CI 7.7–22.0), affecting 13 patients. Non‑neurological complications (infection, hemorrhage, and wrong‑site gyrus resection) are also summarized.
